# Interplay between *Lactobacillus rhamnosus* GG and *Candida* and the involvement of exopolysaccharides

**DOI:** 10.1111/1751-7915.12799

**Published:** 2017-08-03

**Authors:** Camille N. Allonsius, Marianne F. L. van den Broek, Ilke De Boeck, Shari Kiekens, Eline F. M. Oerlemans, Filip Kiekens, Kenn Foubert, Dieter Vandenheuvel, Paul Cos, Peter Delputte, Sarah Lebeer

**Affiliations:** ^1^ Department of Bioscience Engineering Research Group Environmental Ecology and Applied Microbiology University of Antwerp Groenenborgerlaan 171 B‐2020 Antwerp Belgium; ^2^ Department of Pharmaceutical, biomedical and veterinary sciences Laboratory of Pharmaceutical Technology and Biopharmacy University of Antwerp Wilrijk Belgium; ^3^ Department of Pharmaceutical Sciences Natural Products & Food Research and Analysis University of Antwerp Wilrijk Belgium; ^4^ Department of Biomedical Sciences Laboratory of Microbiology Parasitology and Hygiene University of Antwerp Wilrijk Belgium

## Abstract

A number of clinical studies have shown protective effects of lactobacilli against *Candida* species in the gastrointestinal tract, the urogenital tract and the oral cavity, while others did not show clear effects. Evidence on the mode of action of lactobacilli against *Candida* is also still lacking. In this study, the anti‐*Candida* activity of the model probiotic strain *Lactobacillus rhamnosus* GG was explored in different assays to determine molecular interactions. We found that *L. rhamnosus* GG was able to interfere with *Candida* growth, morphogenesis and adhesion. These three aspects of *Candida*'s physiology are all crucial to its opportunistic pathogenesis. In follow‐up assays, we compared the activity of *L. rhamnosus* GG wild‐type with its exopolysaccharide (EPS)‐deficient mutant and purified EPS to evaluate the involvement of this outer carbohydrate layer. Our data demonstrate that purified EPS can both interfere with hyphal formation and adhesion to epithelial cells, which indicates that EPS is part of a combined molecular mechanism underlying the antihyphal and anti‐adhesion mechanisms of *L. rhamnosus* GG.

## Introduction

Under normal circumstances, members of the *Candida* genus are non‐pathogenic, commensal microorganisms inhabiting the gastrointestinal tract, oral cavity and female reproductive tract (Sardi *et al*., 2013). A few species are known to shift to opportunistic pathogens and cause mucosal infections at these sites which in turn can lead to invasive candidiasis. The gastrointestinal tract in particular acts as a frequent source from which *Candida* can disseminate to other tissues and organs (Manzoni *et al*., 2006). For example in critically ill patients, *Candida* species are also regularly cultured from the respiratory tract (Ibàñez‐Nolla *et al*., 2004). The gastrointestinal tract is not the only site where *Candida* can shift to a pathogenic state. Affecting almost 50% of women of reproductive age, vulvovaginal candidiasis (VVC) belongs to the most common forms of *Candida* infections (Foxman *et al*., 2013). The main cause of all these infections is *C. albicans*, but *C. glabrata* has recently gained in importance as a human pathogen (Silva *et al*., 2012). Together, they are responsible for approximately 65%‐75% of all systemic candidiasis infections, which places *C. albicans* and *C. glabrata* currently among the most common fungal pathogens in humans (Brunke and Hube, 2013).

The pathogenesis of *Candida* starts with fungal overgrowth, followed by adhesion, tissue invasion and mucosal infection (Höfs *et al*., 2016). This process is facilitated by several virulence factors including host recognition biomolecules and hyphal morphogenesis. In *C. albicans*, adhesion to epithelial cells is mediated by the agglutinin‐like sequence (*ALS*) gene family proteins, which mainly recognize certain peptides on the host cell surface, but the binding of the key adhesin Als1 to laminin can be inhibited by galactose (Klotz *et al*., 2004; Donohue *et al*., 2011). In *C. glabrata*, three members of the epithelial adhesin (*EPA*) gene family were also found to bind to ligands containing a terminal galactose residue (Zupancic *et al*., 2008). Besides lectin‐like proteins, adhesion of *C. albicans* and *C. glabrata* also seems to involve the oligomannosides on their cell wall surface (Dalle *et al*., 2003; de Groot *et al*., 2008). Hyphal morphogenesis implies the reversible transition between unicellular yeast cells and filamentous growth forms, which is typical for some yeasts such as *C. albicans*, but not *C. glabrata* (Calderone and Fonzi, 2001). The hyphal form of *C. albicans* is associated with alterations in antigen expression and tissue affinities. In addition, hyphae are more potent in penetrating epithelial tissues and causing damage than the unicellular yeast cells (Gow *et al*., 2011).

The human microbiota appears to contribute to the prevention of *Candida* species shifting from a harmless commensal to a disease‐causing pathogen, especially in the gut (Mason *et al*., 2012). In women with VVC, the protective role of the vaginal microbiota in this disease is less clear (Zhou *et al*., 2009; Liu *et al*., 2013). Some studies indicate that lactobacilli, important members of a healthy vaginal microbiota, can protect against vaginal *C. albicans* infections (Martinez *et al*., 2009; Ehrström *et al*., 2010; Kovachev and Vatcheva‐Dobrevska, 2015; Parolin *et al*., 2015), but in another study, the administration of lactobacilli was insufficient to prevent post‐antibiotic vulvovaginitis (Pirotta, 2004). A few studies have also shown protective effects of some lactobacilli, such as *Lactobacillus rhamnosus*, in the oral cavity of elderly (Hatakka *et al*., 2007) and in the gastrointestinal tract of preterm neonates and children (Manzoni *et al*., 2006; Romeo *et al*., 2011; Kumar *et al*., 2013). Proposed modes of action of lactobacilli against *Candida* include immunomodulation of the host epithelial barrier and reduction of *Candida* adhesion by co‐aggregation and through competition for binding sites (Lebeer *et al*., 2010; Rizzo *et al*., 2013; Parolin *et al*., 2015). These beneficial effects of lactobacilli are often considered species‐ and even strain‐specific and have been proposed to involve cell surface components, including peptidoglycan, teichoic acids, (glyco)proteins and polysaccharides (Kleerebezem *et al*., 2010; Lebeer *et al*., 2010), but without further substantiation. One of the best‐documented probiotic strains is *Lactobacillus rhamnosus* GG ATCC 53103 (*L. rhamnosus* GG or LGG). This strain, isolated from a healthy human intestinal microbiota, is one of the probiotic strains with the largest number of documented health benefits, mainly regarding the prevention and recovery of intestinal tract infections (Gorbach, 1996; Doron *et al*., 2005; Segers and Lebeer, 2014). Here, we first explored the inhibitory effect of *L. rhamnosus* GG on *Candida* growth and two important virulence factors, hyphae morphogenesis and adhesion to target epithelial cells. As in both *C. albicans* and *C. glabrata*, lectin‐like adhesins recognizing glycans containing galactose residues have been described (Zupancic *et al*., 2008; Donohue *et al*., 2011), we investigated the role of the galactose‐rich exopolysaccharides (EPS), the major macromolecules of the outer cell wall later of *L. rhamnosus* GG (Lebeer *et al*., 2009), in the anti‐*Candida* activity by comparing wild‐type *L. rhamnosus* GG with its isogenic EPS mutant CMPG5351 and purified EPS. We hypothesize that the presence of galactose‐rich EPS on the surface of LGG can play an important role in its antipathogenic properties against *Candida* species, as it is a strain‐specific, carbohydrate polymer forming a dominant layer on the surface of *L. rhamnosus* GG (Lebeer *et al*., 2009).

## Results

### *Lactobacillus rhamnosus* GG inhibits the growth of *Candida albicans*

**Figure 1:**
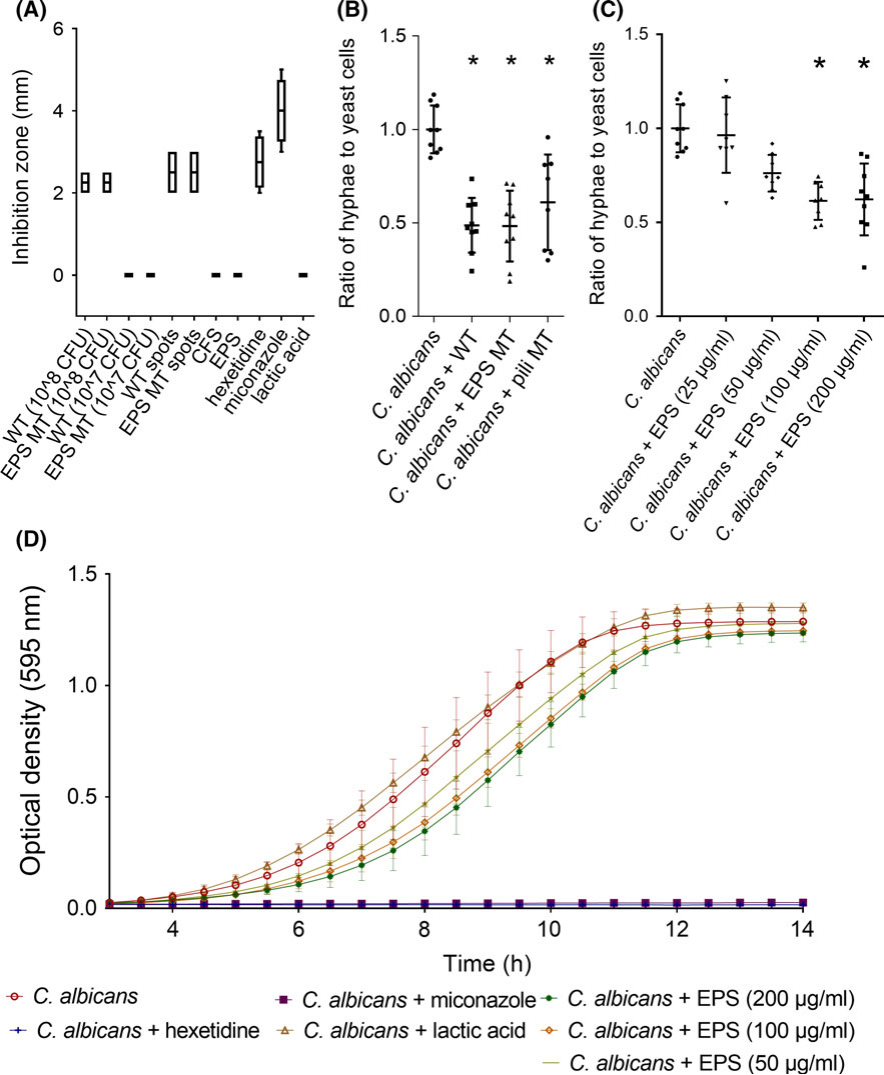
Inhibition of growth and hyphal induction of *Candida albicans*. (A) Well‐diffusion and spot assays of *C. albicans* in the presence of wild‐type *L. rhamnosus* GG (10^8^ CFU ml^−1^), EPS mutant (EPS MT) (10^8^ CFU ml^−1^), CFS, isolated EPS (2 mg ml^−1^) and a 2% L‐lactic acid solution (*n*=4). Hexetidine (0.1%), miconazole (8 μg ml^−1^) were used as positive controls and water as negative control. (B‐C) Hyphal induction of *C. albicans* (10^6^ cells ml^−1^) during co‐incubation with LGG (10^8^ CFU ml^−1^), its EPS mutant (EPS MT) (10^8^ CFU ml^−1^) or isolated EPS. Asterisks indicate p‐values below 0.05 compared to *C. albicans* solely. (D) Evolution of the optical density of *C. albicans* and *C. glabrata* cultures in presence of EPS in different concentrations and a 2% L‐lactic acid solution (*n*=3). Hexetidine (0.1%), miconazole (8 μg ml^−1^) were used as positive controls and water as negative control.

As fungal overgrowth is the initial step in the pathogenesis of *Candida*, we first explored whether the presence of live *L. rhamnosus* GG could control the growth of *C. albicans*. Growth inhibition was investigated using well‐diffusion and spot assays. In well‐diffusion assays, we added overnight cultures of *L. rhamnosus* GG (10^7^ or 10^8^ CFU/ml) or cell‐free supernatant (CFS) of the overnight cultures to wells in agar inoculated with *C. albicans*. In comparison, in spot assays, we added an *C. albicans* inoculated overlay of soft agar on *L. rhamnosus* GG spots. Both *L. rhamnosus* GG added in the wells at 10^8 ^CFU/ml and the *L. rhamnosus* GG spots were able to inhibit *C. albicans* growth (Figure 1A). A ten times lower concentration (10^7^ CFU) and supernatant were insufficient to inhibit *C. albicans* growth. To check how specific for *C. albicans* this inhibition by *L. rhamnosus* GG is, we performed the same assays with *C. glabrata*. This species seemed less sensitive to *L. rhamnosus* GG, as only the spots resulted in growth inhibition (Fig. S1).

To determine possible molecular mechanisms for this growth inhibition, we first tested the effect of lactic acid, as this is one of the major effector molecules of the antimicrobial activity of lactobacilli (De Keersmaecker *et al*., 2006; van den Broek *et al*. submitted). However, lactic acid did not inhibit the growth of *C. albicans* or *C. glabrata* at concentrations comparable to cell‐free culture supernatant of *L. rhamnosus* GG after 24‐h growth, namely 2% L‐lactic acid, as determined with the Roche Yellow line kit (Figure 1A). Subsequently, the influence of EPS from *L. rhamnosus* GG was investigated. The inhibitory capacity of EPS‐deficient strain CMPG5351 in the well‐diffusions and spot assays was comparable to activity of the wild‐type. However, when extracted, purified EPS was able to extend the lag phase in a concentration‐dependent manner. When added to growing *Candida* cultures in a time‐course experiment, we found that isolated EPS (200 μg/ml) prolonged the lag phase of *C. albicans* with approximately 68 minutes (Figure 1D). The EPS concentration range applied in these experiments corresponded with approximately 10^10^ − 4 × 10^10^ CFU/mL *Lactobacillus* cells, which is ca. 100‐fold more than the *Lactobacillus* cultures that interfered with *C. albicans* growth in the well‐diffusion assays. This indicates that other components of *L. rhamnosus* GG are also involved in the direct antimicrobial activity against *C. albicans* activity.

### *Lactobacillus rhamnosus* GG and its isolated EPS molecules inhibit hyphal formation of *Candida albicans*

A crucial virulence factor in the pathogenesis of *C. albicans* is hyphal formation. We examined the effect of *L. rhamnosus* GG on the formation of hyphae by co‐incubating the *C. albicans* yeast cells (10^6^ CFU/ml) with lactobacilli (10^8^ CFU/ml) during hyphal induction with fetal bovine serum (FBS). The presence of *L. rhamnosus* GG was able to reduce the hyphae to yeast cells ratio approximately with 50% (Figure 1B). Furthermore, we could not observe differences in the capacity to reduce *C. albicans* hyphal formation between *L. rhamnosus* GG and its isogenic EPS mutant CMPG5351 (Figure 1B), but remarkably, the isolated EPS was able to reduce the hyphal induction in a concentration‐dependent manner, with a 40% reduction at 100 μg/ml, showing an important role for the EPS molecules in repressing hyphal formation (Figure 1C).

As we observed these effects only for the purified EPS and not for the *welE* mutant of *L. rhamnosus* GG in which specifically galactose‐rich EPS expression was disrupted, we also investigated whether other glycosylated surface components of *L. rhamnosus* GG (Lebeer *et al*., 2012; Tytgat *et al*., 2016) play a role in the antihyphal activity. Hereto, we included the mutant lacking the *spaCBA*‐encoded pili in our experiments (Figure 1B). This mutant was still capable of significantly decreasing hyphal formation of *C. albicans*, to the same extent as *L. rhamnosus* GG wild‐type and the EPS‐deficient *welE* mutant. Elucidating the role of a certain surface component by investigating mutants herein is often complicated by the fact that not only the expression of the target gene is affected, but other surface components can show an altered expression and exposure, as is the case for the *welE* mutant (Lebeer *et al*., 2009, 2012). Therefore, we repeated the antihyphal experiments with EPS isolated from two other *Lactobacillus* strains by the same procedures. These *Lactobacillus* strains, *L. plantarum* CMPG5300 and *L. rhamnosus* GR‐1, have EPS molecules with a different ratio of galactose and glucose in their monomer composition (see Table S1). EPS molecules from these strains were able to inhibit hyphal formation of *C. albicans* to the same extent as EPS from *L. rhamnosus* GG, namely between 45% and 52% at 200 μg/ml (Fig. S2).

### *Lactobacillus rhamnosus* GG and its EPS inhibit adherence of *Candida* to epithelial cells

**Figure 2:**
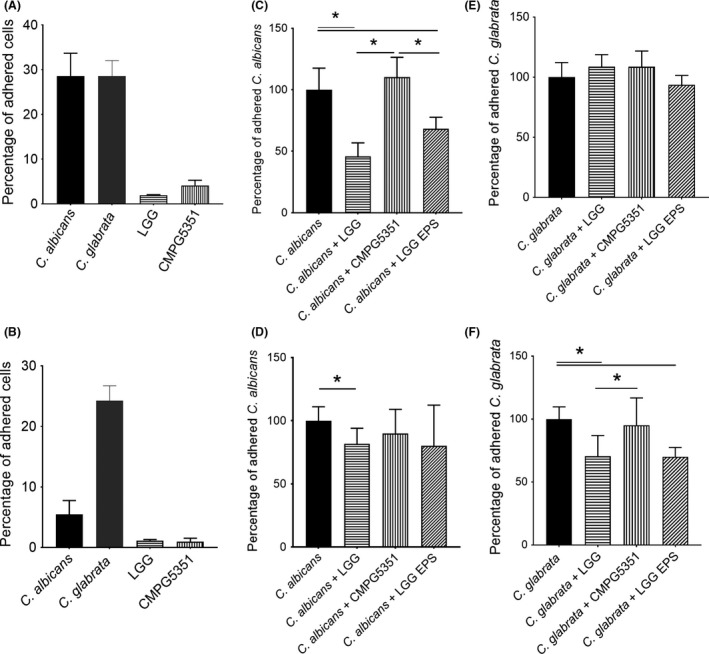
Inhibition of Candida adherence to epithelial cells. (A) Percentage of *Candida albicans*,* C. glabrata*,* Lactobacillus rhamnosus* GG WT and EPS mutant (EPS MT) adhered to VK2/E6E7 monolayers after 1 h of incubation. (B) Percentage of *C. albicans*,* L. rhamnosus* GG and EPS mutant adhered to Calu‐3 monolayers after 1 h of incubation. (C, E) Percentage of *C. albicans* or *C. glabrata* adhered to VK2/E6E7 monolayers when added in competition with *L. rhamnosus* GG WT, EPS mutant or *L. rhamnosus* GG EPS. (D, F) Percentage of *C. albicans* or *C. glabrata* adhered to Calu‐3 monolayers when added in competition with *L. rhamnosus* GG WT, EPS mutant or *L. rhamnosus* GG EPS. The results were normalized to adherence of *C. albicans* solely. In all tests, *C. albicans* and *C. glabrata* were used at an initial concentration of 10^6^ CFU,* L. rhamnosus* GG WT and EPS mutant at 10^8^ CFU, and purified EPS was added to a final concentration of 200 μg ml^−1^. The percentages represent mean values of at least three biological repeats. Asterisks indicate p‐values below 0.05 when conditions were compared to each other.

The following key step in the pathogenesis of *Candida* is its adhesion to epithelial cells. We studied the adhesion of *C. albicans* and *C. glabrata* to both stratified squamous epithelial cells (represented by vaginal cell line VK2/E6E7) and mucous epithelial cells (represented by bronchial cell line Calu‐3). *C. albicans* and *C. glabrata* adhered to both VK2/E6E7 and Calu‐3, although in different amounts. Adherence of both species to VK2/E6E7 cells was approximately 30%, while *C. glabrata* adhered much better to the Calu‐3 cells than *C. albicans* (25% compared with 5%) (Figure 2A and 2B).

To determine the main adhesion inhibition mechanism of *L. rhamnosus* GG, we performed competition, displacement and exclusion assays (Figure 2 and Fig. S3). For the stratified epithelial cells (VK2/E6E7), competition appears to be the main mechanism for *C. albicans* adhesion inhibition by *L. rhamnosus* GG. Upon co‐addition of pathogen and probiotic, we observed a 55% reduction of the adhesion of *C. albicans*. This correlates with a reduction of multiplicity of infection of approximately 2:1 to 1:1 (ration yeast cells: epithelial cells). In contrast, *L. rhamnosus* GG was not able to significantly reduce *C. glabrata* adhesion to these cells. For the mucous epithelial cells (Calu‐3), *L. rhamnosus* GG was able to inhibit *C. albicans* and *C. glabrata* adhesion in both competition and displacement assays.

Next, we investigated whether the EPS layer is of importance for *L. rhamnosus* GG to inhibit *Candida* adhesion. Hereto, we compared the effect of *L. rhamnosus* GG wild‐type on the adhesion of *C. albicans* and *C. glabrata* with its EPS‐deficient mutant strain. For the displacement and exclusion assays with *C. albicans* and *C. glabrata*, no major differences were observed between *L. rhamnosus* GG and the EPS mutant (Fig. S2). However, the EPS mutant CMPG5351 differed remarkably from *L. rhamnosus* GG wild‐type in competition assays, as it was not able to inhibit *C. albicans* nor *C. glabrata* adhesion (Figure 2). We repeated these assays with isolated EPS from *L. rhamnosus* GG instead of the live lactobacilli. Isolated *L. rhamnosus *GG EPS molecules (at 200 μg ml^−1^) were able to reduce the adhesion of *C. albicans* to VK2/E6E7, and to Calu‐3, and of *C. glabrata* to Calu‐3 with approximately 30%, 27% and 25% respectively (Figure 2).

## Discussion

In the present study, we showed that *L. rhamnosus* GG can be used to interfere with growth of *Candida* in *in vitro* experiments. In addition, some important pathogenesis characteristics including adhesion, tissue invasion and mucosal infection were also affected. Our comparison of the activity of wild‐type *L. rhamnosus* GG to its EPS‐deficient mutant CMPG5351 and its purified EPS molecules indicates that its outer EPS layer is – at least partially – involved in its anti‐*Candida* adhesion activity.

*L. rhamnosus* GG was able to interfere with the growth of *C. albicans*, which corresponds with the study of Hasslöf and colleagues, in which they also found inhibitory effects of *L. rhamnosus* GG (from 10^7^ CFU ml^−1^) on *C*. *albicans* growth in one other growth interference test (Hasslöf *et al*., 2010). The inhibition of *C. albicans* by *L. rhamnosus GG* observed in this study was comparable with inhibition by other lactobacilli, but – in contrast to what we expected – the key metabolite lactic acid could not inhibit *C. albicans* growth when added in concentrations comparable to those in *L. rhamnosus* GG supernatant. This is in contrast to a previous study concluding that lactic acid at low pH reduces the metabolic activity of *C. albicans* and eventually kills the cells (Köhler *et al*., 2012). Our other control, miconazole, was more efficient at inhibiting *C. albicans* and *C. glabrata* growth than *L. rhamnosus* GG live cells, but use of this antifungal agent is linked to the development of resistance (Sanglard and Odds, 2002), signifying the need for alternative antifungal strategies. Previously, EPS molecules from *L. plantarum* R315 and kefiran, a branched glucogalactan surrounding kefir, were shown to exhibit anti‐*C. albicans* activity in agar‐diffusion assays at 300 μg ml^−1^ and 450 μg ml^−1^ respectively (Rodrigues *et al*., 2005; Li *et al*., 2014). Our data do not indicate an important role for EPS in the anti‐*Candida* growth activity of *L. rhamnosus* GG, as adding isolated EPS only resulted in prolonging the lag phase. Results of agglutination assays, as described by (Malik *et al*., 2016), indicate that this effect is not caused by *Candida* aggregation by EPS (data not shown). Additional mechanisms in the anti‐*Candida* growth activity of *L. rhamnosus* GG thus seem highly probable. For instance, H_2_O_2_ production has been suggested to have antagonistic properties against *C. albicans* (Strus *et al*., 2005), but this molecule is not highly produced by *Lactobacillus rhamnosus* strains (Felten *et al*., 1999).

More striking than the growth reduction by *L. rhamnosus* GG was its ability to inhibit hyphal formation in *C. albicans*, which is probably the most important virulence step of *C. albicans* as this cell type enables epithelial tissue penetration (Gow *et al*., 2011; Höfs *et al*., 2016). We found that the isolated EPS molecules from *L. rhamnosus* GG on themselves can act as effective inhibitors of hyphal formation, as they reduce the number of hyphae with almost 50%. This effect was also found for the EPS from other *Lactobacillus* strains, having different relative abundances of galactose in their monomer composition, indicating that the activity of EPS from *L. rhamnosus* GG is probably due to its long, complex, galactose‐containing polymer structure, but not solely to its exact strain‐specific sugar composition and configuration.

Intriguingly, we could not observe differences between *L. rhamnosus* GG wild‐type and the galactose‐rich EPS‐deficient mutant CMPG5351, which suggests that other cellular factors could also play a significant role. This could be the glucose‐rich cell wall polysaccharides that are overexposed in the *welE* mutant (Francius *et al*., 2008). Another glycosylated surface factor we investigated were the *spaCBA*‐encoded pili, important for certain molecular interactions of *L. rhamnosus* GG (Lebeer *et al*., 2012; Tytgat *et al*., 2016). The pilus‐deficient mutant also inhibited hyphal formation to same extent as *L. rhamnosus* GG wild‐type and EPS‐deficient mutant, indicating that the pili are not key for this activity. Previously, live lactobacilli, their supernatant and butyric acids have been suggested to interfere with the hyphal induction in *C. albicans* (Noverr and Huffnagle, 2004), but to our knowledge no *Lactobacillus* exopolysaccharides with antihyphal activity have yet been described. The exact mechanism by which EPS reduces hyphal formation forms an interesting topic for further investigation, as this EPS is a potent candidate drug to reduce hyphae and thus virulence of *C. albicans*.

The next key step in the establishment of *Candida* infection is adhesion to the epithelial cells of mucosal surfaces (Höfs *et al*., 2016). It has been suggested that lactobacilli can interfere with adhesion of *Candida* through competition for binding sites or by co‐aggregating with the *Candida* species, involving peptides on the bacterial surface and carbohydrates on the yeast surface as co‐aggregating factors (Ocaña and Nader‐Macías, 2002; Parolin *et al*., 2015; Malik *et al*., 2016). In our results, we observed clear differences in adhesion levels between the two epithelial cell lines and the two tested *Candida* species. This is probably due to different receptor expression on the different epithelial cell lines. Despite the fact that only the major adhesins of *C. glabrata* have galactose‐binding properties, we found that *L. rhamnosus* GG was more efficient in inhibiting adhesion of *C. albicans* than *C. glabrata*, mainly by competition and displacement. Exclusion seems the least important anti‐adhesion mechanism of *L. rhamnosus* GG against *Candida*, which can be explained by the rather low adhesion of *L. rhamnosus* GG itself to these epithelial types.

When we used the isogenic EPS mutant CMPG5351 to evaluate the *in situ* effect of the EPS on anti‐*Candida* adhesion capacity of *L. rhamnosus* GG, we found the intriguing result that this mutant inhibited adhesion far less than *L. rhamnosus* GG in competition assays. Next, we observed that the purified EPS itself was also able to inhibit adhesion in competition assays. To our knowledge, an anti‐*Candida* adhesion capacity for *Lactobacillus* EPS molecules has only been described once before. This study showed that EPS from *L. crispatus* L1 was able to reduce *C. albicans* adhesion to VK2/E6E7 cells, but mainly by exclusion and at ten times higher concentrations (1 mg ml^−1^) (Donnarumma *et al*., 2014) than used in our study. This indicates that the surface polysaccharides of *L. rhamnosus* GG might serve as one of the key molecules for interfering with the binding between the fungal lectin‐like adhesins and host sugars or between the fungal cell wall carbohydrates and their epithelial adhesion receptor, at least in case of direct competition for adhesion.

In conclusion, our data indicate that *L. rhamnosus* GG has potential as adjuvant in antifungal strategies, especially against the major pathogen *C. albicans*, as this probiotic strain inhibits growth, hyphal formation and adhesion. Our reductionist mechanistic approach should, however, be substantiated by future studies considering the role of this probiotic against *Candida* in a microbiota setting, preferentially in clinical trials. In these follow‐up trials, the clinical relevance of the observed decreased hyphal formation and yeast adherence could then be determined. In our pursuit to unravel the molecular mechanisms involved, we found that the EPS layer of *L. rhamnosus *GG might be part of a combined mode of action for the reduction of hyphal formation and for the direct competition with *Candida* during adhesion. Identification of such key effector molecules could substantiate the selection of novel *Lactobacillus* strains with even more effective anti‐*Candida* activity and help explain observed biological effects *in vivo* in clinical trials such as Hatakka *et al*. (2007); Manzoni (2007); Martinez *et al*. (2009); Romeo *et al*. (2011), as well as promote the application of isolated biomolecules.

## Experimental procedures

### Microbial strains and culture conditions

*Lactobacillus* strains (Table 1) were grown at 37 °C without agitation in de Man, Rogosa and Sharpe (MRS) broth (Difco, Erembodegem, Belgium). *Candida* strains (Table 1) were grown while shaking at 37 °C in yeast extract peptone dextrose (YPD) broth (Carl Roth, Karlsruhe, Germany). Hyphal growth of *C. albicans* was induced by supplementing YPD broth with 10% heat inactivated fetal bovine serum (FBS) (Thermo Fischer, Asse, Belgium).

**Table 1:** Bacterial and yeast strains used in this study

Strain	Reference	Description
*Lactobacillus rhamnosus* GG ATCC 53103	ATCC	
*L. rhamnosus* CMPG5351	(Lebeer *et al*., 2009)	Priming enzyme of the production of long galactose‐rich EPS molecules is inactivated
*L. rhamnosus* CMPG5357	(Lebeer *et al*., 2012)	*spaCBA*‐encoded pili are inactivated
*L. rhamnosus* GR‐1 ATCC 55826	ATCC	
*L. plantarum* CMPG5300	(Malik *et al*., 2013)	
*Candida albicans* SC5314	(Fonzi and Irwin, 1993)	
*C. glabrata* ATCC 2001	ATCC	

### Cell culture

The vaginal epithelial cell line VK2/E6E7 ATCC® CRL‐26216™ (purchased from ATCC, Molsheim, France) was maintained at 37 °C with 5% CO_2_ and 90% relative humidity in 75 cm^2^ tissue culture flasks containing serum‐free keratinocyte medium (Life Technologies, Ghent, Belgium) supplemented with CaCl_2_, human epidermal growth factor and bovine pituitary extract. Every 3 days, when the VK2/E6E7 monolayers reached 70%–80% confluency, cells were reseeded with a 1:7 split ratio in fresh culture medium using a 0.25% trypsin‐EDTA solution (Life Technologies). For adhesion and immunomodulation experiments, VK2/E6E7 cells were seeded in 12‐well culture plates (Cellstar, Diegem, Belgium) at a density of 1.6 × 10^5^ cells ml^−1^. Within 3–4 days after seeding, confluent monolayers were obtained. The human bronchial epithelial cell line Calu‐3 ATCC® HTB‐55™ (purchased from ATCC) was cultured in 75‐cm² flasks containing 20 ml Minimum Essential Medium (Life Technologies) supplemented with heat inactivated FBS and penicillin‐streptomycin (100 U ml^−1^) (Life Technologies) and maintained in a humidified 5% CO_2_ incubator at 37 °C. The culture medium was changed every 3–4 days, and the cells were passaged weekly at a 1:2 split ratio using a trypsin‐EDTA solution. For adhesion and immunomodulation experiments, Calu‐3 cells were seeded in 12‐well culture plates at a density of 1.85 × 10^6^ cells ml^−1^. Approximately a week after seeding, confluent monolayers were obtained.

### Isolation and characterization of EPS

The EPS of the *Lactobacillus* strains was isolated with the extraction protocol described previously (Lebeer *et al*., 2007). Briefly, the lactobacilli were grown to an optical density of 0.6 and washed with phosphate‐buffered saline. EPS was then extracted by incubation in 0.05 M EDTA (Sigma‐Aldrich, Diegem, Belgium) (shaking, on ice), followed by ethanol precipitation and dialysis against distilled water [Spectra/Por® dialysis membrane (Spectrum Laboratories, Breda, the Netherlands)]. Afterwards, samples were treated with trichloroacetic acid (Sigma‐Aldrich) to remove proteins, dialysed against water and filter sterilized [pore size 0.2 μm (VWR, Haasrode, Belgium)]. The total amount of carbohydrate was estimated by the phenol‐sulfuric acid method (DuBois *et al*., 1956). Samples were freeze‐dried in a FreeZone 1 Liter Benchtop Freeze Dry System (Model 7740030) (Labconco, MO, USA) and stored at 4°C until use. Before use, the EPS was dissolved in pure water. The purity of the EPS samples after extraction was checked with ^1^H NMR. NMR Spectra were recorded in D_2_O (Sigma‐Aldrich) on a Bruker DRX‐400 instrument, operating at 400 MHz for ^1^H using standard Bruker software. These spectra (shown in Fig. S4) were compared with the spectra described in (Landersjö *et al*., 2002) and indicated in the beginning the presence of some unexpected components, originating from the filters and membranes used during extraction. The isolation protocol was subsequently adapted by rinsing all filters and membranes before use, which resulted in elimination of these compounds. The sugar monomer composition of purified EPS from *L. rhamnosus* GR‐1 and *L. plantarum* CMPG5300 was determined by gas chromatography after hydrolysis and derivatization to alditol acetates (Englyst and Cummings, 1984). Β‐D‐Allose was used as an internal standard, and calibration samples containing the expected monosaccharides were included with each set of samples (Lebeer *et al*., 2009).

### Preparation and determination of lactic acid concentration in supernatant

After overnight incubation, cell‐free culture supernatant was obtained by centrifugation (15 min, 4000 rpm) and filter sterilization (pore size 0.2 μm). The concentration of lactic acid was measured with the commercially available Roche Yellow line kit (Roche, Basel, Swiss).

### Antimicrobial spot‐ and well diffusion‐based agar assays of lactobacilli against *Candida*

Dedicated agar interaction assays for lactobacilli against *Candida* were optimized based on (Schillinger and Lücke, 1989) and (Coconnier *et al*., 1997). Briefly, 2 μl of overnight *Lactobacillus* cultures (approximately 3 × 10^9^ CFU ml^−1^) were spotted on MRS agar (40 ml) and incubated for 48 h. After incubation, YPD soft agar (20 ml) was inoculated with 200 μl of an overnight culture of *C. albicans* or *C. glabrata*, was poured over the agar. After incubation for 24 h, the resulting inhibition zones around the spots were measured. Alternatively, 60 mL YPD agar was inoculated with 2% of an overnight culture of *C. albicans* or *C. glabrata*. After the agar dried, holes (or wells) with a 0.4 cm diameter were made in the agar. Then, 30 μl of *L. rhamnosus* GG cells (10^7^–10^8^ CFU ml^−1^), a 2(m V^−1^)% L‐lactic acid (Carl Roth) solution or purified exopolysaccharides (2 mg ml^−1^), was added to the holes. The plates were incubated for 24 h, and the resulting inhibition zones around the holes were measured. Hexetidine (0.1%) (Johnson & Johnson, Beerse, Belgium), miconazole (8 μg ml^−1^) (Sigma‐Aldrich) and sterile water were used as positive and negative controls respectively.

### Time‐course analysis of the antimicrobial activity of *Lactobacillus* EPS for *Candida* growth in suspension

A time‐course analysis was performed as described previously (De Keersmaecker *et al*., 2006) with minor modifications. Briefly, an overnight culture of *C. albicans* or *C. glabrata* (± 5 × 10^7^ CFU ml^−1^) was added to the wells of a microtiterplate in a 100‐fold dilution, supplemented with *Lactobacillus* EPS (50 μg ml^−1^, 100 μg ml^−1^ and 200 μg ml^−1^). Hexetidine (0.1%), miconazole (8 μg ml^−1^), a 2(m V^−1^)% L‐lactic acid solution and demineralized H_2_O (10%) were used as two positive and the negative control respectively. *Candida* cultures were allowed to grow for 24 h, and the optical density was measured each 30 min at 595 nm using a Synergy HTX multi‐mode reader (Biotek, Drogenbos, Belgium). Each condition was measured at least in triplicate, and the average OD was calculated.

### Inhibition of hyphal formation by *Candida albicans*

*C. albicans* hyphae (10^6 ^CFU ml^−1^) were induced by FBS, while incubated with or without lactobacilli (10^8 ^CFU ml^−1^) or *Lactobacillus* EPS (50 μg ml^−1^, 100 μg ml^−1^ and 200 μg ml^−1^). After incubation, at least one hundred yeast cells and/or hyphae in at least three biological repeats were counted, and the ratio of hyphae to yeast cells was calculated.

### Inhibition of *Candida albicans* and *C. glabrata* adherence to epithelial cells by *Lactobacillus* species

The influence of *L. rhamnosus* GG and CMPG5351 on the adherence of *Candida* species to vaginal epithelial VK2/E6E7 cells and bronchial epithelial Calu‐3 cells was investigated as described previously with minor modifications (Malik *et al*., 2013; Rizzo *et al*., 2013). Three different procedures were used to differentiate between competition between *Candida* and *L. rhamnosus* GG or CMPG5351, exclusion and displacement of *Candida* by *L. rhamnosus* GG or CMPG5351. Competition tests were carried out by adding a volume of 1 ml containing *Candida* cells (10^6^ CFU) and lactobacilli (10^8^ CFU) to tissue culture plate wells containing confluent monolayers of epithelial cells, which were allowed to incubate at 37 °C for 1 h to mediate adherence. For exclusion tests, the monolayers were first incubated with lactobacilli (10^8^ CFU) for 1 h. After incubation, non‐adhered lactobacilli were removed by washing with Dulbecco's PBS (Life Technologies), *C. albicans* cells (10^6^ CFU) were added and incubated for 1 h. For displacement tests, the monolayers were first incubated with *Candida* cells (10^6^ CFU). After incubation of 1 h, the non‐adhered cells were removed by washing with PBS. Afterwards, the lactobacilli were added (10^8^ CFU) to the epithelial cells, and the plates were incubated for another hour. After final incubation, the cells were washed three times with Dulbecco's PBS to remove all non‐adhered cells, and the number of adhered *Candida* and *Lactobacillus* cells to the VK2/E6E7 and Calu‐3 cells was determined by the macrodilution method on Sabouraud agar (Carl Roth), which is selective for fungal species, and MRS agar with cycloheximide (10 mg L^−1^) (Sigma‐Aldrich), which is selective for the lactobacilli. Each condition was carried out at least in triplicate.

### Statistics

Data are presented as mean values ± standard deviation. Shapiro–Wilk normality test (GraphPad Prism 7.00, CA, USA) was used to determine whether the data are normally distributed. Each condition was compared to their negative control with the unpaired Student's *t*‐test (GraphPad Prism 7.00). P‐values below 0.05 were considered to be significant, and significant differences are indicated with asterisks.

## Conflict of Interest

None declared.

## Supporting information


**Fig. S1.** Growth inhibition of C. glabrata.

**Fig. S2.** Inhibition of C. albicans hyphal formation by Lactobacillus EPS.

**Fig. S3.** Inhibition of Candida adherence to epithelial cells by displacement and exclusion.

**Fig. S4.** 400‐MHz 1H NMR spectra of EPS from L. rhamnosus GG before (A) and after (B) protocol optimization, recorded in D2O.

**Table S1.** Monomer composition of Lactobacillus EPS.
