# Supplementary figures and images for: Interplay between Lactobacillus rhamnosus GG and Candida and the involvement of exopolysaccharides
Source: Microb Biotechnol. 2017 Aug 3;10(6):1753–63. doi: 10.1111/1751-7915.12799 (PMC5658588; doi:10.1111/1751-7915.12799)

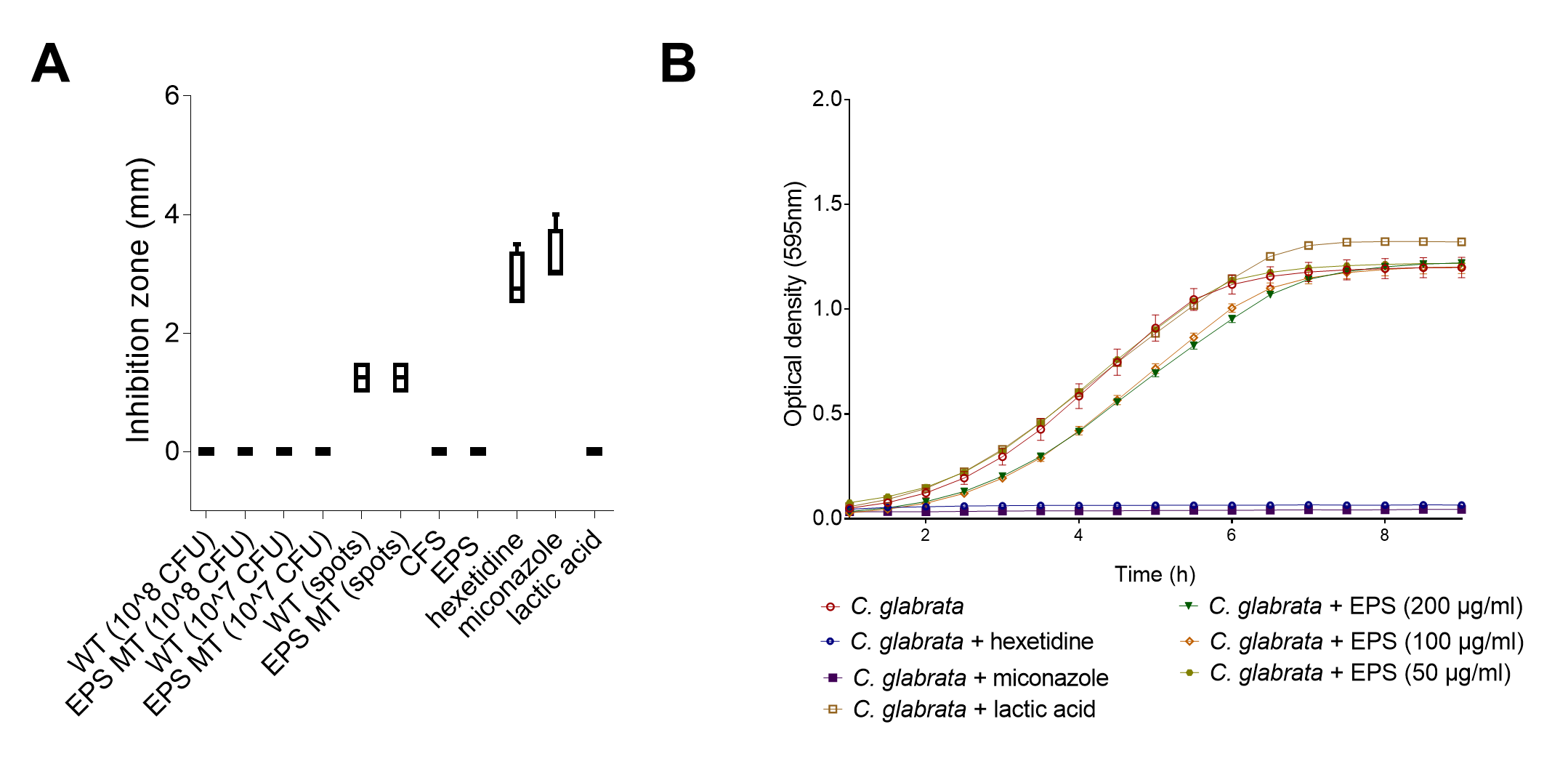

Supplement: Supplementary file 1 — Fig. S1. Growth inhibition of C. glabrata. [file MBT2-10-1753-s001.tif]

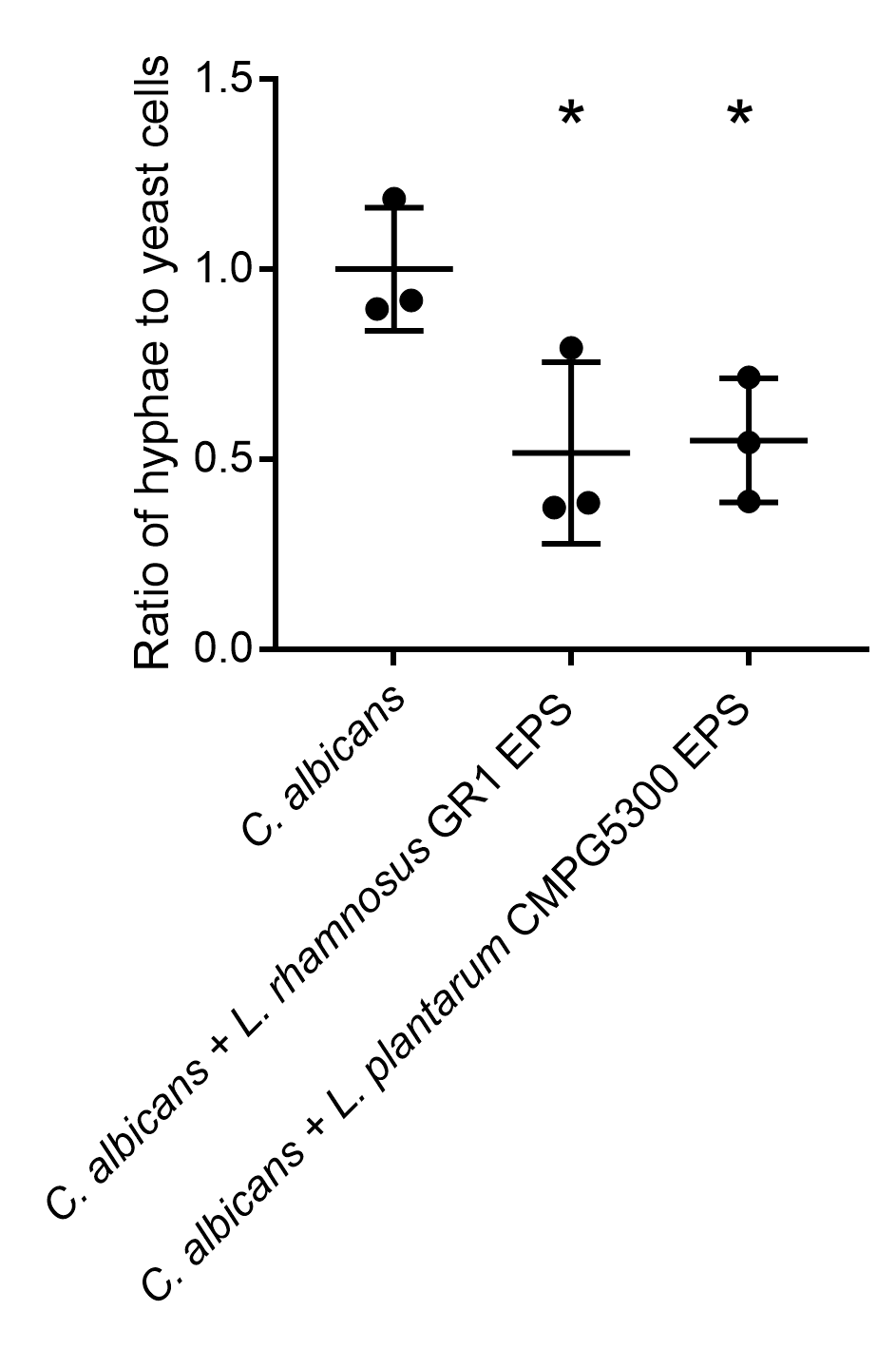

Supplement: Supplementary file 2 — Fig. S2. Inhibition of C. albicans hyphal formation by Lactobacillus EPS. [file MBT2-10-1753-s002.tif]

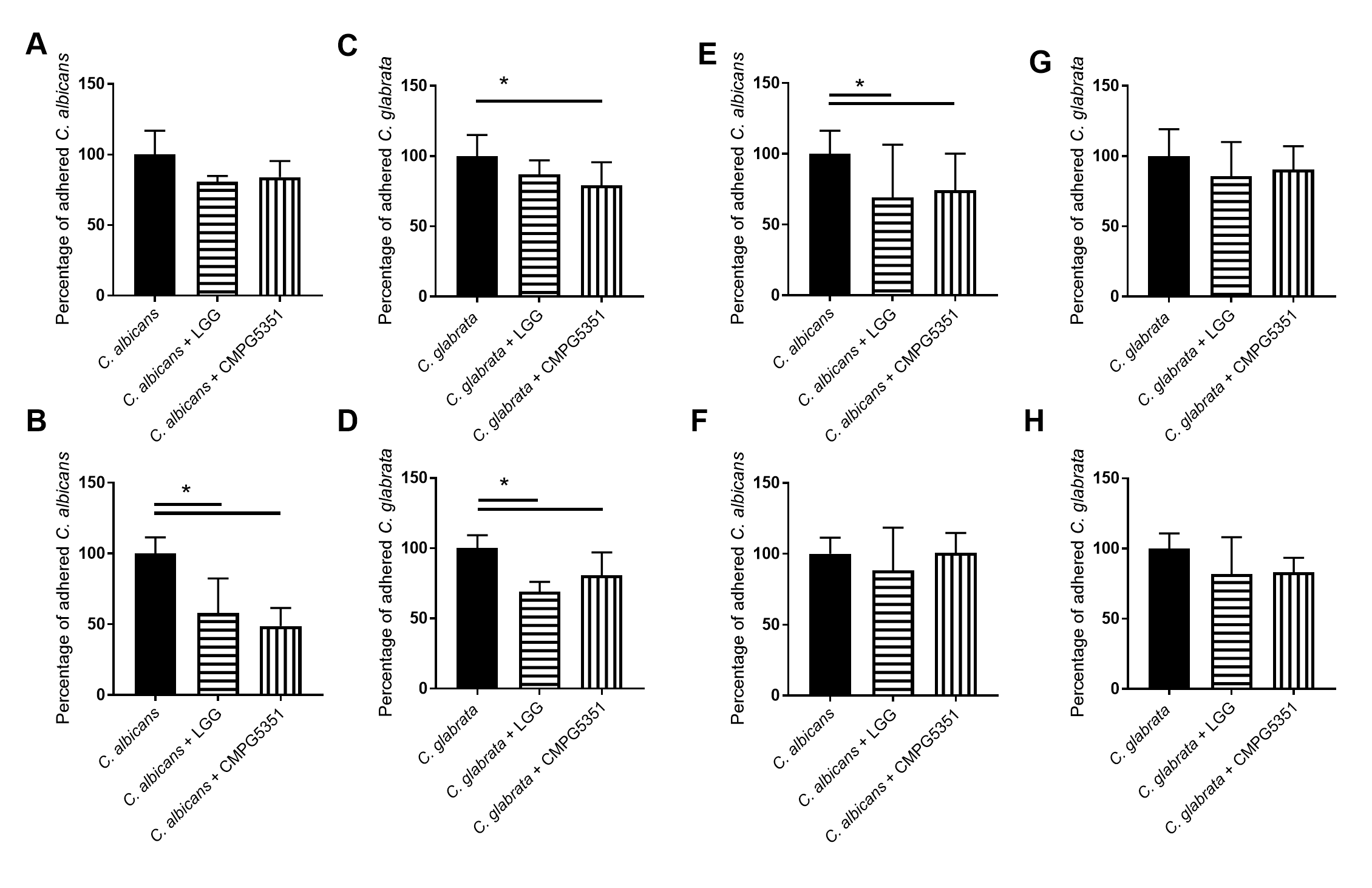

Supplement: Supplementary file 3 — Fig. S3. Inhibition of Candida adherence to epithelial cells by displacement and exclusion. [file MBT2-10-1753-s003.tif]

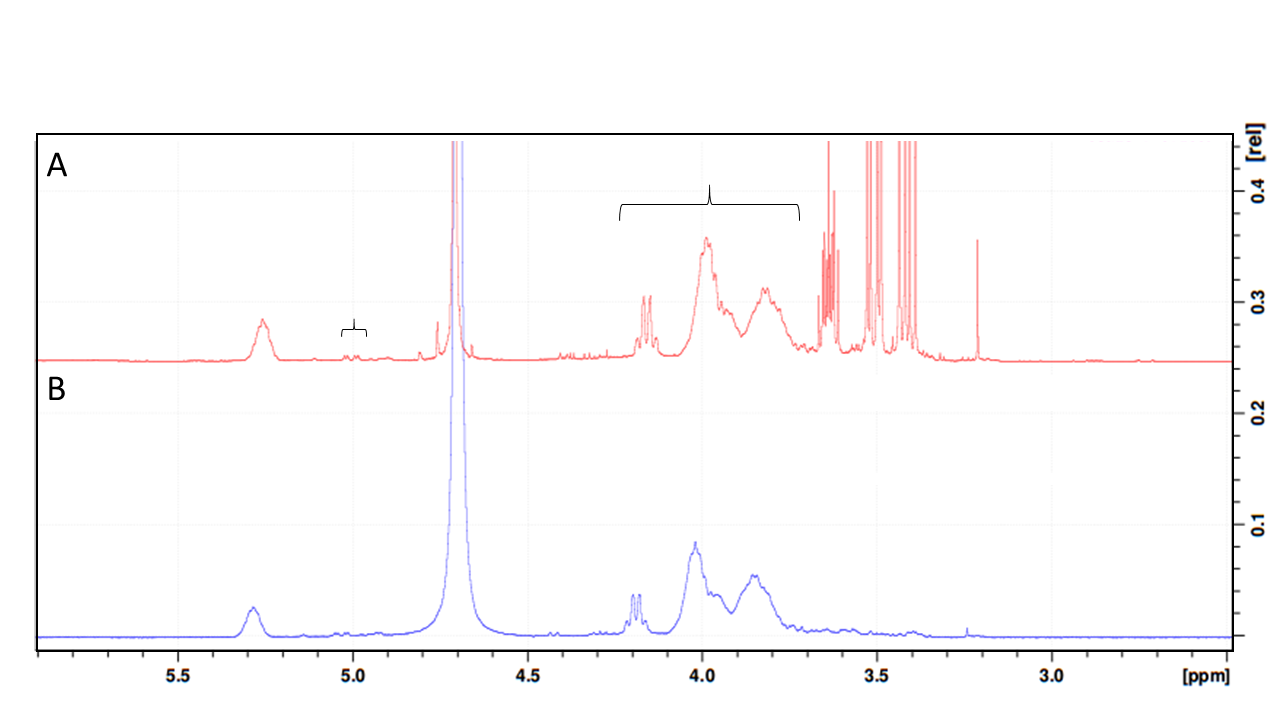

Supplement: Supplementary file 4 — Fig. S4. 400‐MHz 1H NMR spectra of EPS from L. rhamnosus GG before (A) and after (B) protocol optimization, recorded in D2O. [file MBT2-10-1753-s004.tif]
