# Supplementary material for: Interplay between Lactobacillus rhamnosus GG and Candida and the involvement of exopolysaccharides
Source: Microb Biotechnol. 2017 Aug 3;10(6):1753–63. doi: 10.1111/1751-7915.12799 (PMC5658588; doi:10.1111/1751-7915.12799)
Supplement: Supplementary file 5 — Table S1. Monomer composition of Lactobacillus EPS. [file MBT2-10-1753-s005.docx]

Strain D-glucose D-galactose D-mannose D-xylose L-arabinose L-rhamnose

*L. rhamnosus* GR-1 81.84% 18.52% 0% 0% 0% 0%

*L. plantarum* CMPG5300 77.63% 10.53% 6.58% 0% 5.26% 0%
